# Procalcitonin-guided antibiotic therapy in patients with congestive heart failure and suspicion of lower respiratory tract infection: results from a randomized trial

**DOI:** 10.1186/cc11969

**Published:** 2013-03-19

**Authors:** P Schuetz, E Grolimund, A Kutz, S Haubitz, B Mueller

**Affiliations:** 1 Kantonsspital Aarau, Switzerland

## Introduction

Differentiation of acute heart failure from infection in patients with respiratory symptoms and a history of congestive heart failure (CHF) is challenging due to overlap of clinical symptoms and X-ray findings. The BACH study found higher mortality rates if patients presenting with dyspnea were treated with antibiotics and their procalcitonin (PCT) levels were low indicating absence of bacterial infection. Yet the BACH study was observational and causal inference cannot be drawn. Herein, we analyzed the effects of PCT-guided antibiotic stewardship in CHF patients from a previous trial (ProHOSP).

## Methods

This is a secondary analysis of a previous randomized trial of adult ED patients with respiratory symptoms and a history of CHF. Patients were randomized to administration of antibiotics based on a PCT algorithm (PCT group) or standard guidelines without knowledge of PCT levels (control group). The primary endpoint of this analysis is the risk of adverse outcome defined as death or ICU admission within 30 days after ED admission.

## Results

**Figure 1:**
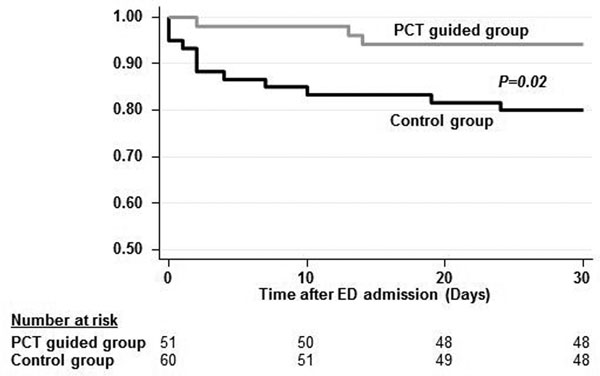
**Time to adverse outcome according to group allocation**.

A total of 233 patients met the inclusion criteria, with 116 in the PCT-guided group and 117 in the control group. In the subgroup of patients with low initial PCT levels <0.25 ng/l (*n *= 110), PCT-guided patients had a significant reduction in antibiotic exposure (mean 3.7 vs. 6.5 days, difference -2.8 (95% CI -4.4, -1.2), *P <*0.001). Furthermore, PCT-guided patients had a significant lower risk for death and ICU admission (4% vs. 20%, odds ratio 6.0 (1.3, 28.2), *P *= 0.02). See Figure 1.

## Conclusion

In CHF patients with suspicion of respiratory infection, use of a PCT protocol resulted in a significant decrease of antibiotic exposure and significantly improved outcomes in patients with low PCT levels indicating absence of bacterial infection. Whether inadequate antibiotic therapy in these CHF patients requiring diuretic treatment explains this difference in clinical outcomes needs verification.

